# Visualizing locus-specific sister chromatid exchange reveals differential patterns of replication stress-induced fragile site breakage

**DOI:** 10.1038/s41388-019-1054-5

**Published:** 2019-10-21

**Authors:** Irina Waisertreiger, Katherine Popovich, Maya Block, Krista R. Anderson, Jacqueline H. Barlow

**Affiliations:** 1 Department of Microbiology and Molecular Genetics, University of California, Davis, CA 95616 USA; 2 Genome Center, University of California, Davis, CA 95616 USA

**Keywords:** Homologous recombination, Double-strand DNA breaks, Chromosomes

## Abstract

Chromosomal fragile sites are genomic loci sensitive to replication stress which accumulate high levels of DNA damage, and are frequently mutated in cancers. Fragile site damage is thought to arise from the aberrant repair of spontaneous replication stress, however successful fragile site repair cannot be calculated using existing techniques. Here, we report a new assay measuring recombination-mediated repair at endogenous genomic loci by combining a sister chromatid exchange (SCE) assay with fluorescent in situ hybridization (SCE-FISH). Using SCE-FISH, we find that endogenous and exogenous replication stress generated unrepaired breaks and SCEs at fragile sites. We also find that distinct sources of replication stress induce distinct patterns of breakage: ATR inhibition induces more breaks at early replicating fragile sites (ERFS), while ERFS and late-replicating common fragile sites (CFS) are equally fragile in response to aphidicolin. Furthermore, SCEs were suppressed at fragile sites near centromeres in response to replication stress, suggesting that genomic location influences DNA repair pathway choice. SCE-FISH also measured successful recombination in human primary lymphocytes, and identificed the proto-oncogene *BCL2* as a replication stress-induced fragile site. These findings demonstrate that SCE-FISH frequency at fragile sites is a sensitive indicator of replication stress, and that large-scale genome organization influences DNA repair pathway choice.

## Introduction

Replication stress is a potent source of DNA breaks in proliferating cells and is frequently elevated in cancer cells [1, 2]. Disruptions in replication fork stability generate replication stress, leading to increased fork stalling or collapse. Specific genomic regions called fragile sites are exquisitely sensitive to replication stress, accumulating high levels of DNA breaks in response to chemical or genetic perturbations of DNA replication [3, 4].

Common fragile sites (CFS) were identified as sites of recurrent DNA breaks in cells exposed to the DNA polymerase inhibitor aphidicolin (APH). CFSs primarily occur in gene-poor, late replicating regions enriched for AT repeats prone to forming secondary structures [5–7]. We recently identified a new class of fragile sites occurring in gene-rich regions with a high density of replication origins termed early replicating fragile sites (ERFS) [8]. ERFS are transcriptionally active, and are enriched for CpG islands—a common feature of mammalian promoters. Studies of fragile site stability directly measure unsuccessful DNA repair using fluorescent in situ hybridization (FISH) to visualize DNA breaks in metaphase chromosome spreads. Though CFSs and ERFSs have distinct genetic and epigenetic features, FISH studies revealed that oncogene overexpression and ATR inhibition induce frequent DNA breaks at both sites in primary B cells [6, 8, 9].

Collapsed replication forks contain a double-strand break (DSB) intermediate, and homologous recombination (HR) plays a critical role in fork recovery. Cells lacking the HR factors Brca1, Rad51, Xrcc2, or Mus81 exhibit increased DNA breaks at fragile sites, suggesting that HR suppresses spontaneous replication stress-associated damage [8, 10–12]. Xrcc2 is a Rad51 paralog, forming a heterotetrameric complex with its family members Rad51B, Rad51C, and Rad51D (BCDX2) [13]. This complex stimulates HR and influences the choice between short and long-tract gene conversion [14].

Unrepaired fragile site breaks are readily detected following induced replication stress. However, it is not known if fragile sites experience spontaneous replication stress that is normally repaired, as no prior studies measured successful DNA repair at fragile sites on the single cell level. DNA blotting and PCR amplification-based measurements of successful recombination have limited utility at fragile sites [15]. Both techniques require mapping break sites within 2–20 kilobases (kb), while fragile sites span >100 kb [16, 17]. In addition, only ~10% of cells contain damage at an individual fragile site, further hampering detection by these methods.

Here we combine sister chromatid exchange (SCE) with FISH—SCE-FISH—to measure successful HR-mediated repair at endogenous fragile sites in mouse and human primary lymphocytes. We found that replication stress from inhibition of either ATR or DNA polymerase induced DNA breaks and SCEs at ERFSs and CFSs in WT and HR-deficient *Xrcc2*^*f/f*^ mouse B cells. Further, SCE-FISH revealed that Xrcc2 is not required for replication stress-induced SCE formation. We also observed distinct differences in SCE frequency at ERFSs and CFSs in response to ATRi and APH, indicating that exogenous sources of replication stress differentially affect early and late-replicating fragile sites. We also investigate the effects of genomic location on fragile site stability and repair pathway choice.

## Results

### SCE-FISH measures locus-specific DNA repair

**Fig. 1:**
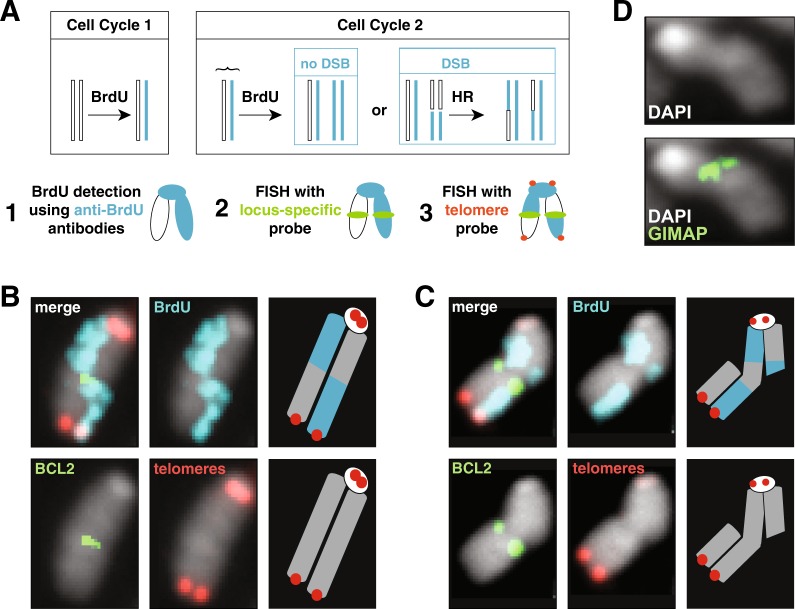
SCE-FISH measures successful recombination-mediated repair at endogenous genomic loci. **a** SCE-FISH assay scheme. SCE is an event where the two strands of DNA exchange after repair of a DSB, resulting in a crossover event. SCEs can be visualized by differentially labeling the two sister chromatids with the nucleotide analog BrdU. Combining single locus FISH with BrdU staining to measure SCE events allows the measurement of successful DSB repair at a specific locus on a single cell level. Telomere probe to visualize chromosome ends facilitates cytogenetic analysis of DNA damage. FISH probes are shown in green, telomere-specific probe is in red, and BrdU shown in cyan. **b** SCE-FISH validation showing a spontaneous SCE at the ERFS locus *BCL2*. **c** Chromatid break at the fragile site *BCL2*. **d** Example of a spontaneous DNA break at *GIMAP* in *Xrcc2*^*f/f*^ B cells. *GIMAP* is in green, DAPI in greyscale. Probe for fragile sites in green, telomeres in red, BrdU in cyan, and DAPI in greyscale. Images in **c** and **d** taken from cells exposed to 1 μM ATRi

HR-mediated repair involves invasion of the adjacent sister chromatid to prime new DNA synthesis. The resulting cruciform structure—the Holliday junction—can be resolved as noncrossover or crossover events, the latter generating SCEs. SCEs are visualized through the differential labeling of sister chromatids by incorporating the nucleoside analog bromodeoxyuridine (BrdU) into DNA for two rounds of replication (Fig. 1a). To simultaneously visualize SCEs and single-locus FISH, we detected BrdU by immunofluorescent staining (Fig. 1a). Unlike immuno-FISH involving protein detection, the bromine-modified thymidine analog recognized by the BrdU antibody is heat, protease, and formamide-insensitive, yielding robust and repeatable fluorescent signal when combined with standard FISH procedures (Fig. 1b, c). In addition, SCE-FISH helped visualize mitotic chromosome damage; BrdU staining helped differentiate between chromosomes harboring chromatid breaks from twisted but intact sister chromatids (Fig. 1c, Supplementary Fig. 1a).

### SCE-FISH reveals spontaneous DNA repair at endogenous fragile sites

To measure DNA damage and repair at individual fragile sites, we performed SCE-FISH in antigen-stimulated WT and XRCC2-deficient mouse primary B cells undergoing rapid proliferation [18]. We measured breaks and SCEs at two ERFSs (*GIMAP* and *BCL2*), two CFSs (*IMMP2L* and *FHIT*), and two control loci termed cold sites (64O1 and 164J15)—chosen for their distance from mapped fragile sites (>15 MB). *Xrcc2*^*f/f*^ cells act as a positive control, as ~10% of metaphases contain DNA breaks compared with 0–2% in wild type cells [8], and damage at *GIMAP* is a frequent event (Fig. 1d, Supplementary Fig. 1b, c).

**Fig. 2:**
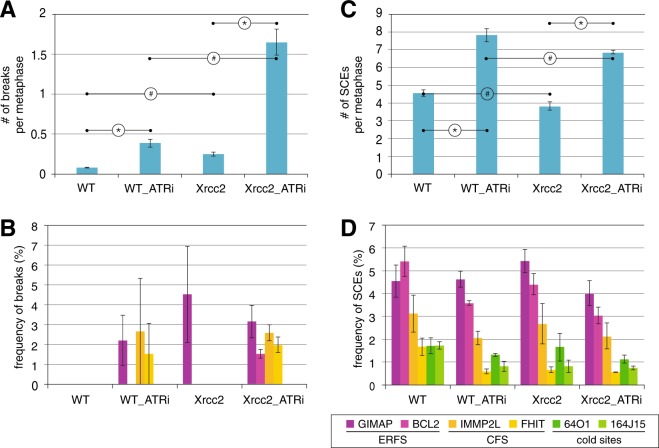
Exposure to 1 μM ATR inhibitor induces DNA breaks and SCE events at ERFSs and CFSs. **a** Number of DNA aberrations per metaphase in response to 1 μM ATRi in WT and *Xrcc2*^*f/f*^ cells. 1 μM ATRi induces an average of 0.4 breaks/cell in WT and 1.7 breaks/cell in *Xrcc2*^*f/f*^ cells. **b** Frequency of DNA aberrations at individual ERFSs, CFSs, and cold sites. **c** Number of SCEs per metaphase in response to 1 μM ATRi in WT and *Xrcc2*^*f/f*^ cells. Untreated WT cells harbor an average of 4.6 SCE/cell, and *Xrcc2*^*f/f*^ cells contain 3.8 SCE/cell. **d** Frequency of SCEs at individual ERFSs, CFSs, and cold sites. Error bars show the standard error of mean (SEM) from three independent experiments. Statistics: *p < 0.05 comparing untreated and ATRi-treated cells for each genotype; ^#^*p* < 0.05 comparing WT and *Xrcc2*^*f/f*^ cells treated with ATRi. All break frequencies with standard error and pairwise *P* values for individual loci are provided in Supplementary Table 1. For each independent FISH experiment, B cells were isolated and cultured from a separate mouse. A minimum of 50 metaphase spreads were analyzed for each experiment, resulting in a minimum of 150 metaphases analyzed

In the absence of exogneous replication stress, WT cells contained virtually no DSBs (<0.01 breaks/metaphase), and no breaks at fragile or cold sites (Fig. 2a, c). *Xrcc2*^*f/f*^ cells harbored ~0.25 breaks/metaphase with ~4% of breaks at the ERFS *GIMAP*; these were the only spontaneous fragile site or cold site breaks observed (Fig. 2a, b). In contrast to DSBs, we observed extensive spontaneous SCE formation (Fig. 2c). Similar to previous reports, we observed 15% fewer spontaneous SCEs in *Xrcc2*^*f/f*^ cells than WT cells [19] (Fig. 2c). Both WT and *Xrcc2*^*f/f*^ cells harbored 2.5-fold more SCEs at the ERFSs *GIMAP* and *BCL2* than cold sites, and 1.8-fold more SCEs at the CFS *IMMP2L* (Fig. 2d). Intriguingly, the SCE frequency at *FHIT* was similar to cold sites. These results suggest that *GIMAP*, *BCL2*, and *IMMP2L* experience more spontaneous DNA damage and recombination than cold sites.

### ATR inhibition induces DNA damage and recombination at ERFSs and CFSs

To measure replication stress-induced fragile site breakage, we analyzed DNA aberrations in WT and *Xrcc2*^*f/f*^ cells exposed to a small molecule inhibitor of ATR, ETP-46464 (ATRi). The DNA damage checkpoint kinase ATR is a central player in the replication stress response, and loss of ATR activity leads to replication-associated genome instability and cell death [20–22]. Approximately 2% of WT cells contained breaks at *GIMAP*, *IMMP2L*, and *FHIT* in response to 1 μM ATRi, however total damage (0.4 breaks/cell) was too low to calculate break frequency accurately at individual loci. Therefore, we measured ATRi-induced damage in *Xrcc2*^*f/f*^ cells where breaks were 3.5-fold higher (Fig. 2a). All four fragile sites co-localized with DNA damage in 2.5–3% of *Xrcc2*^*f/f*^ cells, compared with no breaks at cold sites [8, 23] (Fig. 2b, Supplementary Fig. 2b).

To calculate the rate of successful repair, we next analyzed SCE formation. In response to 1 μM ATRi, total SCEs increased 1.5-fold in WT and *Xrcc2*^*f/f*^ cells (Fig. 2c). The number of SCEs also increased at *GIMAP*, *BCL2*, and *IMMP2L—*however the relative frequency of SCE formation at fragile sites was comparable between ATRi-treated and untreated cells (Fig. 2d, Supplementary Table 1). These results support the hypothesis that ATR inhibition induces genome instability by impeding the cellular response to spontaneous replication stress. In addition, the SCE frequency at individual fragile sites was similar in WT and *Xrcc2*^*f/f*^ cells in the presence or absence of ATRi (Fig. 2d). Together, these results indicate that Xrcc2 is not required for spontaneous and ATRi-induced SCE formation at fragile sites.

Similar to untreated cells, SCEs at *FHIT* were fourfold lower than *IMMP2L* in ATRi-treated cells (Fig. 2d, Supplementary Table 1). This difference is highlighted by SCE frequency within the cell population: 15% of ATRi-treated WT cells have an SCE at *IMMP2L* while fewer than 5% have an SCE at *FHIT* (Supplementary Fig. 2d). We observed similar levels of damage at *FHIT* and *IMMP2L* (Fig. 2b, Supplementary Table 1, Supplementary Fig. 2b), therefore these results raise the possibility that SCE formation is suppressed at *FHIT*.

### Increasing ATRi concentration enhances fragile site damage

**Fig. 3:**
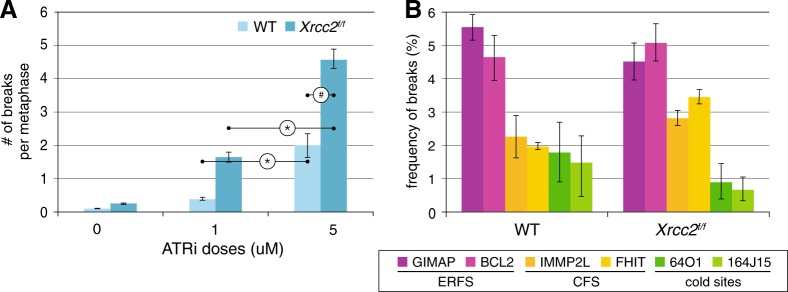
5 μM ATR inhibitor induces high level of DNA breaks at ERFS. **a** Number of DNA aberrations per metaphase in response to 5 μM ATRi treatment. We observed an average of 2 breaks/cell in WT cells and 4.4 breaks/cell *Xrcc2*^*f/f*^ cells. **b** Frequency of DNA aberrations at ERFSs, CFSs, and cold sites. Error bars show SEM from three independent experiments. Statistics: *p < 0.05 comparing untreated and ATRi treated cells for each genotype; ^#^*p* < 0.05 comparing WT and *Xrcc2*^*f/f*^ cells exposed to ATRi. All break frequencies with standard error and pairwise *P* values for individual loci are provided in Supplementary Table 2

One micromolar ATRi induces modest levels of damage in WT cells; therefore, we increased the drug concentration to confirm that ERFSs and CFSs are sensitive to ATRi. Compared with 1 μM ATRi, 5 μM ATRi increased total DNA damage 5-fold in WT cells and 2.5-fold in *Xrcc2*^*f/f*^ cells (Fig. 3a). Exposure to 5 μM ATRi led to distinct differences in the frequency of fragile site breaks in WT and *Xrcc2*^*f/f*^ cells. ERFS harbored extensive damage in WT and XRCC2-deficient cells—breaks at *GIMAP* or *BCL2* comprised of ~5% of total aberrations (Fig. 3b, Supplementary Table 2). In contrast, CFS breaks occurred more frequently than cold sites only in *Xrcc2*^*f/f*^ cells (~3% vs. ~1% of total damage). Thus, ERFSs are more sensitive to ATRi-induced replication stress than CFSs.

Intriguingly, 5 μM ATRi exposure increased the number of cells with DNA breaks at both fragile site alleles, revealing that replication fork stress occurred at the same locus on both chromosomes (Supplementary Table 2). This finding raises the possibility that the lack of an intact repair template prevents inter-homolog recombination, resulting in unrepaired breaks in mitosis.

### ERFSs are hotspots of aphidicolin-associated DNA damage

**Fig. 4:**
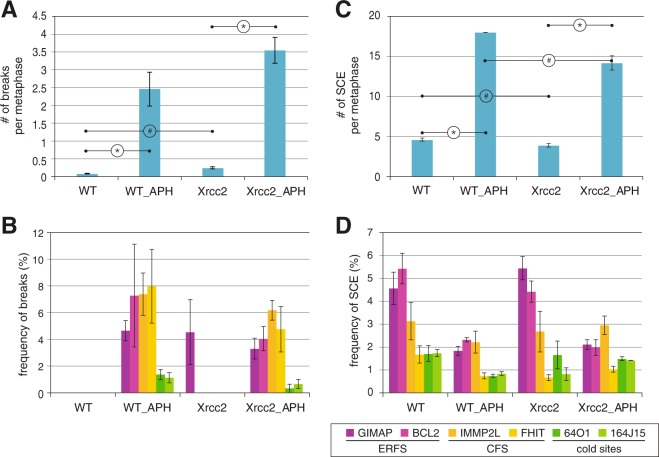
Exposure to aphidicolin induces DNA breaks and SCE events at ERFSs and CFSs. **a** Number of DNA aberrations per metaphase in response to APH in WT and *Xrcc2*^*f/f*^ cells. **b** Frequency of DNA damage at ERFSs, CFSs, and cold sites. **c** Number of SCEs per metaphase in response to APH in WT and *Xrcc2*^*f/f*^ cells. APH induces 18 SCE/cell in WT cells, and 14.2 SCE/cell in *Xrcc2*^*f/f*^ cells. **d** Frequency of SCE formation at ERFSs, CFSs and cold sites. Error bars show the standard error of mean (SEM) from three independent experiments. Statistics: *p < 0.05 comparing untreated and APH treated cells of each genotype; ^#^*p* < 0.05 com*p*aring WT and *Xrcc2*^*f/f*^ cells exposed to APH. All break frequencies with standard error and pairwise *P* values for individual loci are provided in Supplementary Table 3

ERFSs and CFSs have contrasting epigenetic and genetic features [24], therefore distinct sources of replication stress may differentially impact ERFS and CFS stability. To test this, we exposed cells to the B-family DNA polymerase inhibitor aphidicolin (APH) which hinders replication initiation and progression [25]. Exposure to 0.4 μM APH induced extensive DNA damage in WT and *Xrcc2*^*f/f*^ cells (Fig. 4a). In WT cells, ~6% of aberrations occurred at the CFSs *FHIT* and *IMMP2L* compared with ~1% of aberrations co-localizing with cold site probes (Fig. 4b, Supplementary Fig. 3a, b). We observed a similar break frequency for the ERFS *GIMAP* and *BCL2* in WT cells, contrary to previous experiments [8]. APH also induced more aberrations in Xrcc2-deficient cells, however this result was not statistically significant. To confirm *Xrcc2*^*f/f*^ cells were hypersensitive to APH, we re-expressed wild-type murine XRCC2 (MIGR1-X2) in *Xrcc2*^*f/f*^ cells by retroviral infection and measured fragile site DSBs. APH-induced DNA damage decreased over 50% in *Xrcc2*^*f/f*^ cells complemented with XRCC2 than cells infected with empty vector (MIGR1-EV) (Supplementary Fig. 3e). However *Xrcc2* re-expression did not affect fragile site breakage; *GIMAP* and *IMMP2L* breaks occurred at the same frequency in MIGR1-EV and MIGR1-X2-expressing cells (Supplementary Fig. 3f).

To measure APH-associated repair, we next analyzed SCEs. APH induced a 3.5-fold increase in total SCEs in WT and *Xrcc2*^*f/f*^ cells (Fig. 4c). SCEs also occurred more frequently at *GIMAP, BCL2*, and *IMMP2L* than cold sites in WT and XRCC2-deficient cells (Fig. 4d). Unlike ATRi, we found no difference in SCE frequency between ERFSs and CFSs—SCEs at all three sites were twofold higher than cold sites (Fig. 4d, Supplementary Table 3). However the SCE frequency at fragile sites was significantly lower than in untreated or ATRi-treated cells (Fig. 4d). WT and *Xrcc2*^*f/f*^ cells had a similar SCE frequency, indicating that XRCC2 is not required for replication stress-induced SCE formation. We propose that APH provokes replication fork stalling throughout S phase, affecting both early and late-replicating fragile sites.

Similar to ATRi, we observed few SCEs at *FHIT* in response to APH—while 25–30% of cells harbor one or more SCEs at *IMMP2L*, <15% had an SCE at *FHIT* (Fig. 4d, Supplementary Fig. 3d). However ~15% of cells contained damage at *FHIT—*similar to other fragile sites (Supplementary Fig. 3b, Supplementary Tables 1, 3). These results further support the hypothesis that replication stress-induced SCEs are suppressed at *FHIT*.

### SCEs are suppressed at centromere-proximal fragile sites

From yeast to humans, centromeres experience meiotic crossover suppression that can affect adjacent genes [26, 27]. *FHIT* is located 7 Mb from the centromere, raising the possibility that it experiences centromere-associated crossover suppression. To test this, we performed SCE-FISH at *IKZF1*, an ERFS located ~7 Mb from the chromosome 11 centromere. In response to 0.4 μM APH, the break frequency at *IKZF1* was twofold higher than cold sites (Supplementary Fig. 4a, Supplementary Table 3). Similar to *FHIT, IKZF1* harbored APH-induced SCEs at cold site levels (Supplementary Fig. 4b, Supplementary Tables 1, 3). In addition, SCEs at *IKZF1* occurred at cold site levels in response to 1 μM ATRi (Supplementary Table 3, Supplementary Fig. 4b). The break frequency of *IKZF1* in response to 1 μM ATRi was too infrequent to measure accurately in WT cells, therefore we exposed cells to 5 μM ATRi. Here, *IKZF1* harbored extensive DNA damage—the break frequency was similar to the ERFS *GIMAP* and *BCL2* (Supplementary Table 2, Supplementary Fig. 4b). We were unable to measure fragile site SCEs in 5 μM ATRi-treated cells due to the low mitotic index; BrdU-labeled metaphases were insufficient. Taken together, this data suggests that SCE formation is suppressed at fragile sites proximal to centromeres.

### Replication stress induces fragile site breaks on both alleles

ERFSs replicate early, suggesting that damaged forks persist many hours to be observed in mitosis. One possible explanation for this persistence is both chromosomes experience damage leaving no intact template for repair. We found evidence for such events: in WT cells exposed to 5 μM ATRi, 4/21 cells with *GIMAP* damage contained breaks at both alleles (Supplementary Table 2c). Further, 10.3% of cells contained an SCE on both *BCL2* alleles, and 3.7% at *GIMAP* (Supplementary Table 1e). HR shows a strong preference to use the sister chromatid in mammals, however the homologous chromosome is also utilized in allelic repair [28]. If both repair templates are damaged, then DSBs may persist into mitosis. The majority of HR-mediated repair events result in noncrossover products, therefore we are likely underestimating this phenomenon. We propose that damage at both alleles—and the absence of a viable repair template—accounts for a significant portion of persistent fragile site breaks observed in mitosis.

### ATRi and APH induce distinct rearrangement types

**Fig. 5:**
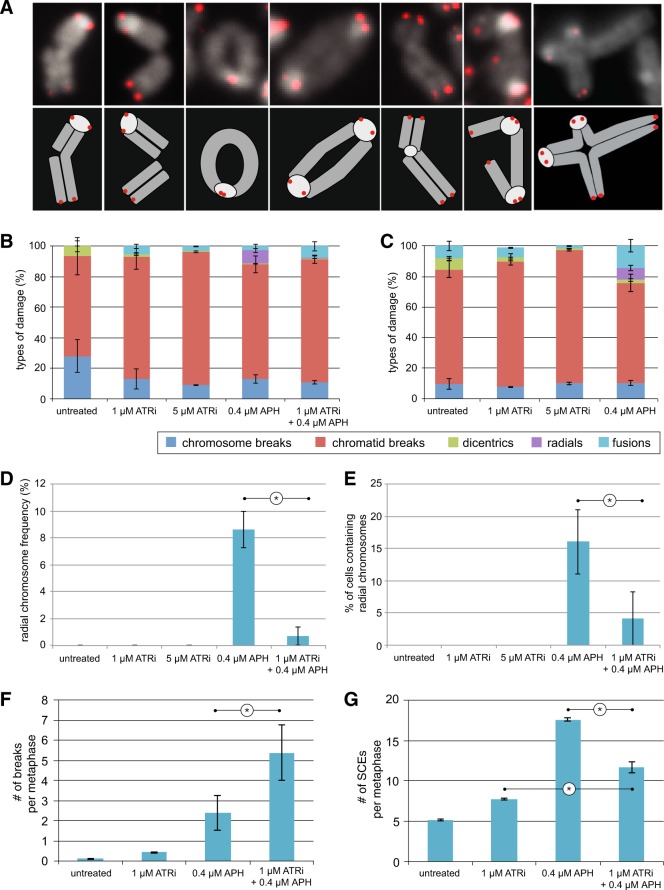
ATRi and APH exposure induce distinct types of DNA aberrations. **a** Representative images of the types of rearrangements produced. Telomere-specific probe visualized in red, DAPI is in greyscale. **b** Frequency of DNA damage types caused by different replication stress treatments in WT cells. **c** Frequency of DNA damage types caused by different treatments in *Xrcc2*^*f/*f^ cells. **d** Frequency of radial chromosomes (% of total damage) in response to different treatments. **e** Percent of cells containing radial chromosomes in response to different treatments. **f** Number of DNA aberrations per metaphase in response to combination of 1 μM ATRi and 0.4 μM APH in WT cells. **g** Number of SCEs per metaphase in response to combination of 1 μM ATRi and 0.4 μM APH in WT cells. Error bars show the SEM from three independent experiments. Statistics: *p < 0.05 comparing differentially treated cells

Both ATRi and APH induce dicentric chromosomes, chromosome breaks, and chromatid breaks—the last comprises over 70% of observed damage (Fig. 5a–c). APH also induces the formation of radial chromosome fusions while ATRi does not (Fig. 5b–e). APH induces more unrepaired breaks than1 μM ATRi; therefore it is possible that having multiple exposed DNA ends in a single cell promotes radial formation (Figs. 2c and 4c). However exposure to 5 μM ATRi dramatically increases the number of unrepaired breaks without inducing radials (Fig. 3a, Fig. 5d, e), therefore increasing DNA breaks is insufficient to drive radial formation. Further, *Xrcc2*^*f/f*^ cells harbor high levels of DNA damage yet contain no radial chromosomes in response to either 1 μM or 5 μM ATRi (Fig. 3a, Fig. 5d, e). Thus, a high level of DNA damage is not sufficient to induce radial formation.

### ATR activity promotes APH-induced radial fusion formation

ATRi and APH both induce replication stress, however radial chromosomes only form in response to APH. To define the impact of ATR inhibition on APH-induced radial chromosome formation, we characterized DNA aberrations and SCE formation in cells exposed to both 1 μM ATRi and 0.4 μM APH (ATRi + APH). As expected, total DNA aberrations were higher in ATRi + APH-treated cells than single treatments (Fig. 5f). ATRi + APH induced a lower rate of total SCEs per cell than APH alone (Fig. 5g), suggesting that ATR activity is required for a subset of SCE events. Neither SCE nor break frequency were significantly different at fragile sites in ATRi + APH-treated cells compared with single treatments (data not shown). Intriguingly, radial chromosomes were greatly reduced in response to ATRi + APH treatment compared with APH alone (Fig. 5f, g).

**Fig. 6:**
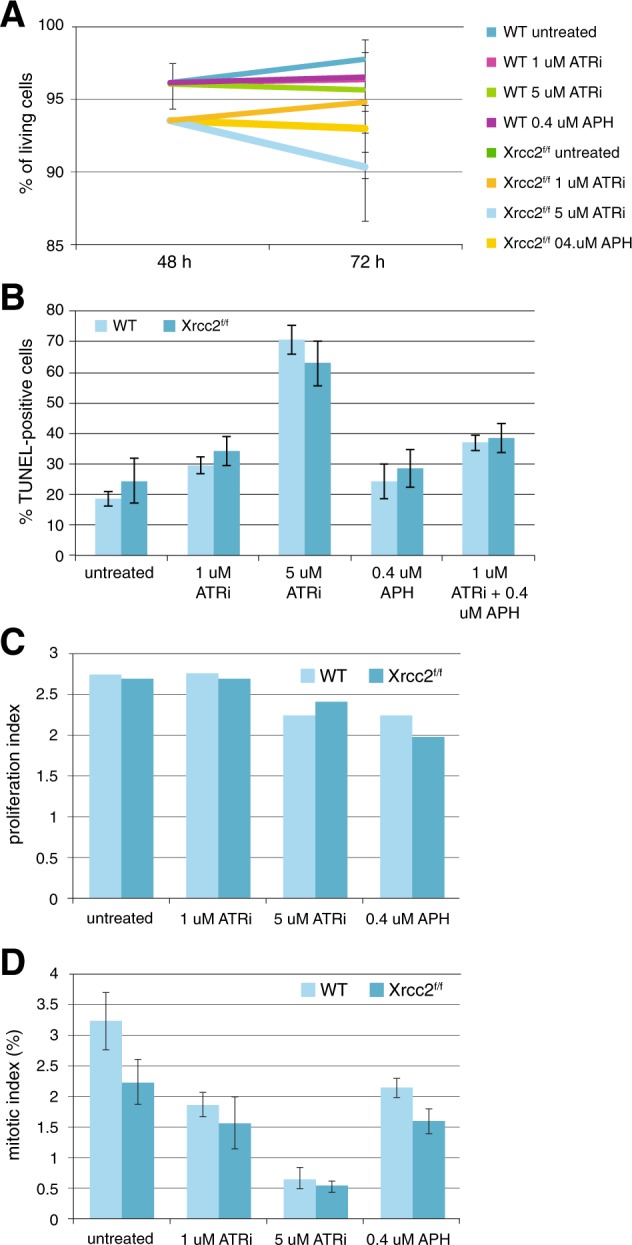
WT and *Xrcc2*^*f/f*^ cells exhibit elevated cell death in response to replication stress. **a** Viability of WT and *Xrcc2*^*f/f*^ cells in response to ATRi and APH treatment measuring cell fragmentation by flow cytometry. **b** Percent of TUNEL-positive WT and *Xrcc2*^*f/f*^ cells in response to ATRi, APH, and combination of ATRi and APH treatment. Error bars show the SEM from three independent experiments. **c** Proliferation index of of WT and *Xrcc2*^*f/f*^ cells in response to ATRi and APH. **d** Mitotic index of WT and *Xrcc2*^*f/f*^ cells in response to ATRi and APH

Radials are potentially cytotoxic DNA rearrangements; chromosome fusions containing more than one centromere promote mitotic errors [29, 30]. Therefore it is possible combined treatment with APH and ATRi leads to increased apoptosis, complicating the analysis of radial formation. To test this, we measured cell viability in ATRi- and APH-treated cells. No treatment increased cell death more than 10% by propidium iodide staining (Fig. 6a). Similarly, the fraction of TUNEL-positive cells modestly increased in response to ATRi + APH (40% vs. 25–30%, Fig. 6b). In contrast, we found that 60% of cells were TUNEL-positive in 5 μM ATRi-treated cells—a twofold increase from 1 μM ATRi (Fig. 6b). Thus, it is unlikely that the lack of radial chromosomes observed in ATRi + APH-treated cells is due to increased cell death (Fig. 6b). These results indicate that ATR kinase activity is required for radial chromosome formation.

### SCE-FISH is elevated at fragile sites in human cells

**Fig. 7:**
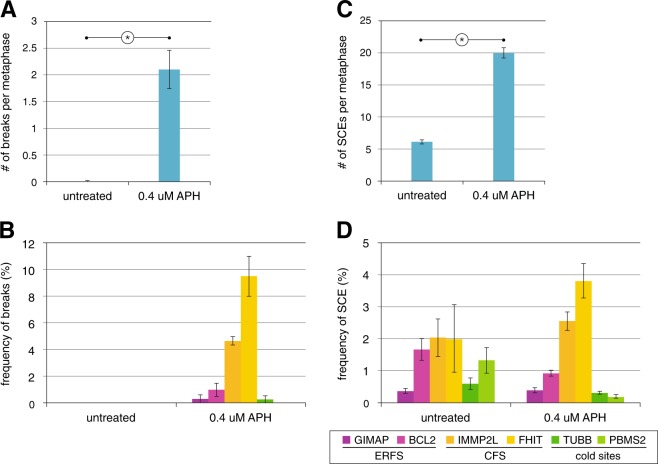
SCE-FISH reveals CFS breakage and repair in primary human peripheral blood lymphocytes. **a** Number of DNA breaks per metaphase in response to 0.4 μM APH. **b** Frequency of DNA aberrations at ERFSs, CFSs and cold sites. **c** Number of SCEs per metaphase in response to 0.4 μM APH. **d** Frequency of SCEs at ERFSs, CFSs, and cold sites. Error bars show the SEM from three independent experiments. *p < 0.05 comparing untreated and 0.4 μM APH-treated cells. For each independent experiment, human peripheral blood specimens obtained from separate donors were used. A minimum of 50 metaphase spreads were analyzed for each experiment, resulting in a minimum of 150 metaphases analyzed

CFSs were first identified in primary human lymphocytes exposed to APH [3, 4]. To measure successful fragile site repair in primary human cells, we exposed stimulated peripheral blood mononuclear cells (PBMC) from whole blood to 0.4 μM APH for 20 h then performed SCE-FISH. Similar to previous reports, APH treatment induced breaks and SCEs in human PBMCs [31] (Fig. 7a, c). APH induced a high level of DNA aberrations, particularly at CFSs (Fig. 7b, Supplementary Fig. 5b). We also observed breaks at the ERFS *BCL2*, albeit at lower levels than either CFS (Fig. 7b; Supplementary Fig. 5b; Supplementary Table 4). APH also increased SCEs at CFSs and ERFSs relative to cold sites (Fig. 7d, Supplementary Table 4). SCEs were strongly elevated at CFSs, correlating with DNA breakage. Nearly 70% of cells harbored an SCE at *FHIT*, compared with ~14% in mouse (Supplementary Fig. 5d). The *FHIT* region shares high sequence homology between mouse and human, however it is located ~30 Mb from the centromere in humans [32]. We hypothesize that chromosomal location drives the difference in SCE rate between human and mouse rather than sequence variation. ERFS also exhibited elevated SCE formation in response to APH; SCEs at *BCL2* were threefold higher than cold sites (Fig. 7d). Together, these results demonstrate that replication stress generates DNA damage at ERFSs and CFSs in human peripheral lymphocytes, and CFSs are significantly more prone to APH-induced damage than ERFSs.

## Discussion

Fragile sites were discovered over 30 years ago, leading to the hypothesis that fragile site instability promotes cancer initiation and development. Indeed, fragile site instability is observed in many human cancers [8, 33–35]. While multiple studies revealed that fragile sites are hypersensitive to exogenous replication stress, assessing their instability in unperturbed cells has remained elusive.

Using SCE-FISH to measure successful fragile DNA repair, we found that spontaneous DNA damage and repair at fragile sites frequently occurs in proliferating cells. Furthermore, exposure to ATRi and APH elicit distinct responses in ERFS and CFS breakage rates. ERFSs harbored more SCEs than CFSs in untreated and ATR inhibitor-treated cells, suggesting that early replicating fragile sites experience more HR-repaired spontaneous damage than late-replicating counterparts. However, ERFSs and CFSs experience elevated damage in response to APH, indicating that perturbation of pol α primase disturbs fork stability in early- and late-replicating regions equally. It will be interesting to determine if these differences arise solely from replication timing, or are governed by additional factors such as transcriptional activity.

HR preferentially repairs transcriptionally active euchromatin while condensed heterochromatin favors NHEJ, suggesting that defective HR would preferentially increase ERFS breaks. However, ERFS and CFS break frequency was similar in *Xrcc2*^*f/f*^ and WT cells. *Xrcc2*^*f/f*^ cells exhibited a modest but reproducible reduction in spontaneous and replication stress-induced SCEs, however SCE frequency at fragile sites is similar to WT cells. These results show that XRCC2 suppresses replication stress-associated instability, however it is largely dispensable for replication stress-induced SCE formation. XRCC2 promotes HR and replication fork protection [36–38], therefore the increase in unrepaired damage in response to ATRi is likely a combination of increased fork collapse and reduced noncrossover repair.

SCE formation at the two fragile sites located near centromeres—CFS *FHIT* and ERFS *IKZF1*— was similar to cold sites in mouse B cells. We did not observe differential BrdU staining at centromeric heterochromatin (Fig. 1b, c); therefore it is possible that SCEs at *FHIT* and *IKZF1* were not detected. However this explanation is unlikely: SCEs were visible at both *FHIT* and *IKZF1* even in highly compacted chromosomes (Supplementary Fig. 4d). We hypothesize that SCEs are suppressed at *FHIT* and *IKZF1* due to their proximity to the centromere, similar to crossover suppression in meiosis [26, 39]. In yeast, the Ctf19 complex promotes cohesion enrichment in the pericentromeric region, suppressing break formation and crossover formation [40]. In mouse, loss of the histone methyltransferase DNMT1 or DNMT3A and DNMT3B leads to increased SCE within centromeres—demonstrating that epigenetic modifications also regulate crossover formation in pericentromeric repeats [41]. Further studies employing SCE-FISH will define whether mitotic SCE suppression is governed by similar or distinct mechanisms.

Different sources of replication stress also induced distinct types of DNA damage. ATRi and APH both generated chromosome and chromatid breaks, however APH also induced radial chromosome fusions. Both ERFS and CFS probes frequently localized at radial fusion junction sites, indicating they are both rearrangement hotspots. We hypothesize that APH exposure generates specific DNA structures that promote radial formation. Radial chromosomes contain multiple centromeres, and have profound effects on genome stability. Multiple centromeres promote severe chromosome segregation defects, mitotic defects, and entry into “breakage-fusion-bridge” (BFB) cycle. Importantly, BFB cycles are implicated in driving oncogene amplification and tumorigenesis in multiple cancers [42–44]. Intriguingly, we found that ATR kinase activity promoted APH-induced radial chromosome formation. It will be interesting to determine if ATR inhibition impacts the viability of cells experiencing BFB.

Fragile sites have emerged as replication stress-specific sites of DNA damage and are exquisitely sensitive to a range of genotoxic agents. HR efficiently repairs spontaneous fragile site damage, therefore why does replication stress induce such a profound increase in unrepaired damage at these sites? Two possibilities likely contribute: (1) fragile sites experience more fork collapse, and (2) the resulting DNA breaks are difficult to repair. Fragile sites are enriched for repetitive DNA, prone to forming secondary structures, and associate with RNA:DNA hybrid (R loop) formation [4, 45, 46]. All three can perturb replication fork progression, and often require additional enzymes for break resolution [47–49].

Here we show that SCE-FISH reveals the ongoing spontaneous DNA damage and successful repair occurring at ERFSs and CFSs in mouse and human B cells. For non-centromeric loci, enhanced SCE formation directly correlates with increases in DNA breaks observed in mitosis—whether they are induced by genetic defects in DNA repair or the application of chemical agents. We predict that high levels of crossovers measured by SCE-FISH at fragile sites can act as a biomarker for patients with high risk for developing second cancers or proliferative syndromes.

## Materials and methods

### Mice and cells

All experiments were performed in accordance with protocols approved by the UC Davis Institutional Animal Care and Use Committee (IACUC protocol #20042). Mice used in this study include *CD19*^*cre*^ and *Xrcc2*^*f/f*^ [50, 51]. Splenic B cells were isolated using the Dynabeads untouched CD43 mouse B cell isolation kit (Thermo Fisher, 11422D) and cultured as previously described [8]. Human lymphocytes were obtained from peripheral blood of three unrelated volunteers and cultured for 72 h in MF-Chang medium (Irvine Scientific, 91005), supplemented with 10% heat inactivated fetal bovine serum and 3 μg/mL phytohemagglutinin (Remel, Inc., 30852701).

### Metaphase chromosome preparation

To visualize SCE, 1 μM BrdU (Sigma, B5002) was added to medium for 20–40 h, depending on cell cycle length. Cells were arrested in metaphase by a 1-h treatment with 0.1 μg/ml demecolcine (Sigma, D1925), treated with 0.075 M KCl, fixed in methanol:acetic acid (3:1), spread onto glass slides and air-dried.

### Drug treatments

ATRi (mTOR Inhibitor XIII, ETP-46464, Millipore, 5.00508.0001) or APH (Fisher Scientific, BP615-1) were added to the cell culture medium 20 h prior to harvest at the designated concentration.

### Bacterial artificial chromosome probes

All Bacterial Artificial Chromosomes (BACs) used for custom-designed probes were purchased from Children’s Hospital Oakland Research Institute Resource Center (BACPAC). Probes were direct-labeled using a nick translation kit (Abbott Molecular, Inc., 07J00-001) with DY-495-dUTP (Dyomics, 495-34) and hybridized to metaphase cell preparations of a karyotypically normal donor to confirm correct mapping prior to experimentation.

### FISH and FISH-SCE

FISH and FISH-SCE studies were performed on metaphase cells using probes described (Supplementary Table 5). A total of 200 ng of each probe were hybridized to target DNA and blocked with ~15-fold excess of human COT DNA (Roche, 11581074001) and salmon sperm DNA (Ambion, AM9680). Prior to hybridization, slides were briefly heated over an open flame, denaturing DNA for BrdU detection. Slides were pretreated at 72 °C in 2 × SSC for 2 min, washed in 1 × PBS at room temperature (RT) for 5 min, post-fixed in 1% formaldehyde at RT for 5 min, and washed in 1 × PBS at RT for 5 min. Slides were dehydrated in ethanol (75, 85, and 100%) at RT for 2 min each and air-dried. Cells and probes were co-denatured at 75 °C for 3 min and incubated overnight at 37 °C in a humid chamber. Slides were washed post-hybridization in 0.4 × SSC/0.3% NP-40 at 72 °C (2 min), then 2 × SSC/0.1% NP-40 at RT (2 min). Slides were probed with 0.25 μM telomere probe (PNA Bio, F1002) for 2 h at RT. Slides were then washed in 1 × PBST (1X PBS, 0.5% Triton-X-100) three times for 5 min at 37 °C. BrdU detection: the primary mouse-anti-BrdU (BD, 347580; 1:200) and secondary Cy5 goat-anti-mouse antibodies (Invitrogen, A10524; 1:200) were used, then washed in 1 × PBST (1X PBS, 0.5% Triton-X-100) three times for 5 min at 37 °C. Slides were counterstained with Vectashield mounting medium containing DAPI (Vector laboratories Inc., H-1200).

### Microscopy and analysis

B cells were isolated and cultured from a separate mouse for each experiment. A minimum of 50 metaphases were analyzed for each experiment. Metaphases images were acquired using an epifluorescent Nikon microscope with NIS Elements AR4.40.00 software (Nikon). Downstream analysis used ImageJ32 software (NIH).

### Statistics

Statistical significance of differences was estimated by Student’s-criterion. To determine if distinct sites have significantly different SCE or break frequencies, we compared individual loci using pairwise analysis. To estimate the correlation between co-occurrence of ERFS and CFS in individual cells, the Cochran–Mantel–Haenszel test was used.

### TUNEL

TUNEL assay was performed using the In Situ Cell Death Detection Kit (Roche, 11684795910). At least 100 nuclei per experiment were analyzed by microscopy. At least three independent experiments were performed for each data set. The statistical significance of differences was estimated by Student’s-criterion.

### Viability assay

Live cells were rinsed twice in 1× Hanks’ Balanced Salt Solution (HBSS) (Gibco, 14065-056), then incubated in 1xHBSS supplemented with 2 mg/ml propidium iodide (Invitrogen, P1304MP) for 10 min at RT. Fluorescence-activated cell sorting (FACS) analysis was carried out on a Becton Dickinson CantoII flow cytometer (BD Biosciences). Up to 20,000 live cells were analyzed for each condition, and data analysis was performed using FlowJo 8.8.32 software.

### Retroviral preparation and B cell infection

Viral supernatants were produced by co-infection of HEK293T cells with MIGR1 (a gift from Warren Pear (Addgene plasmid #27490) and pCL-ECO (a gift from Inder Verma (Addgene plasmid #12371) 72 h before infection. B cell infection was performed as described in [52]. Viral supernatant supplemented with polybrene (2.5 μg/ml) and HEPES (20 mM) was added to cells at 24 and 48 h post-stimulation with RP105/LPS/IL-4. Cells were spinoculated at 2500 RPM for 90 minutes. After 4 h at 37 C, viral supernatant was replaced with B cell media with RP105/LPS/IL-4. At 96 h, GFP + cells were collected by flow cytometry, then harvested for FISH.

## Supplementary information

Supplementary Figure Legends

Supplementary Figure 1

Supplementary Figure 2

Supplementary Figure 3

Supplementary Figure 4

Supplementary Figure 5

Supplementary Table 1

Supplementary Table 2

Supplementary Table 3

Supplementary Table 4

Supplementary Table 5

## References

[1] Berti M, Vindigni A (2016). Replication stress: getting back on track. Nat Struct Mol Biol.

[2] Zeman MK, Cimprich KA (2014). Causes and consequences of replication stress. Nat Cell Biol.

[3] Glover TW, Berger C, Coyle J, Echo B (1984). DNA polymerase alpha inhibition by aphidicolin induces gaps and breaks at common fragile sites in human chromosomes. Hum Genet.

[4] Durkin SG, Glover TW (2007). Chromosome fragile sites. Annu Rev Genet.

[5] Ozeri-Galai E, Lebofsky R, Rahat A, Bester AC, Bensimon A, Kerem B (2011). Failure of origin activation in response to fork stalling leads to chromosomal instability at fragile sites. Mol Cell.

[6] Zlotorynski E, Rahat A, Skaug J, Ben-Porat N, Ozeri E, Hershberg R (2003). Molecular basis for expression of common and rare fragile sites. Mol Cell Biol.

[7] Letessier A, Millot GA, Koundrioukoff S, Lachages AM, Vogt N, Hansen RS (2011). Cell-type-specific replication initiation programs set fragility of the FRA3B fragile site. Nature.

[8] Barlow JH, Faryabi RB, Callen E, Wong N, Malhowski A, Chen HT (2013). Identification of early replicating fragile sites that contribute to genome instability. Cell.

[9] Jiang Y, Lucas I, Young DJ, Davis EM, Karrison T, Rest JS (2009). Common fragile sites are characterized by histone hypoacetylation. Hum Mol Genet.

[10] Ying S, Minocherhomji S, Chan KL, Palmai-Pallag T, Chu WK, Wass T (2013). MUS81 promotes common fragile site expression. Nat Cell Biol.

[11] Arlt MF, Xu B, Durkin SG, Casper AM, Kastan MB, Glover TW (2004). BRCA1 is required for common-fragile-site stability via its G2/M checkpoint function. Mol Cell Biol.

[12] Schwartz M, Zlotorynski E, Goldberg M, Ozeri E, Rahat A, le Sage C (2005). Homologous recombination and nonhomologous end-joining repair pathways regulate fragile site stability. Genes Dev.

[13] Yonetani Y, Hochegger H, Sonoda E, Shinya S, Yoshikawa H, Takeda S (2005). Differential and collaborative actions of Rad51 paralog proteins in cellular response to DNA damage. Nucleic Acids Res.

[14] Nagaraju G, Hartlerode A, Kwok A, Chandramouly G, Scully R (2009). XRCC2 and XRCC3 regulate the balance between short- and long-tract gene conversions between sister chromatids. Mol Cell Biol.

[15] Zhou Y, Paull TT (2015). Direct measurement of single-stranded DNA intermediates in mammalian cells by quantitative polymerase chain reaction. Anal Biochem.

[16] Helmrich A, Stout-Weider K, Hermann K, Schrock E, Heiden T (2006). Common fragile sites are conserved features of human and mouse chromosomes and relate to large active genes. Genome Res.

[17] Rozier L, El-Achkar E, Apiou F, Debatisse M (2004). Characterization of a conserved aphidicolin-sensitive common fragile site at human 4q22 and mouse 6C1: possible association with an inherited disease and cancer. Oncogene.

[18] Lyons AB, Parish CR (1994). Determination of lymphocyte division by flow cytometry. J Immunol Methods.

[19] Deans B, Griffin CS, O’Regan P, Jasin M, Thacker J (2003). Homologous recombination deficiency leads to profound genetic instability in cells derived from Xrcc2-knockout mice. Cancer Res.

[20] Flynn RL, Zou L (2011). ATR: a master conductor of cellular responses to DNA replication stress. Trends Biochemical Sci.

[21] Gaillard H, Garcia-Muse T, Aguilera A (2015). Replication stress and cancer. Nat Rev Cancer.

[22] Cimprich KA, Cortez D (2008). ATR: an essential regulator of genome integrity. Nat Rev Mol Cell Biol.

[23] Lopez-Contreras AJ, Specks J, Barlow JH, Ambrogio C, Desler C, Vikingsson S (2015). Increased Rrm2 gene dosage reduces fragile site breakage and prolongs survival of ATR mutant mice. Genes Dev.

[24] Mortusewicz O, Herr P, Helleday T (2013). Early replication fragile sites: where replication-transcription collisions cause genetic instability. EMBO J.

[25] Baranovskiy AG, Babayeva ND, Suwa Y, Gu J, Pavlov YI, Tahirov TH (2014). Structural basis for inhibition of DNA replication by aphidicolin. Nucleic Acids Res.

[26] Talbert PB, Henikoff S (2010). Centromeres convert but don’t cross. PLoS Biol.

[27] Lambie EJ, Roeder GS (1988). A yeast centromere acts in cis to inhibit meiotic gene conversion of adjacent sequences. Cell.

[28] Richardson C, Moynahan ME, Jasin M (1998). Double-strand break repair by interchromosomal recombination: suppression of chromosomal translocations. Genes Dev.

[29] Gascoigne KE, Cheeseman IM (2013). Induced dicentric chromosome formation promotes genomic rearrangements and tumorigenesis. Chromosome Res.

[30] McClintock B (1941). The stability of broken ends of chromosomes in Zea Mays. Genetics.

[31] Porfirio B, Dallapiccola B, Gandini E (1985). The effect of aphidicolin on Fanconi’s anemia lymphocyte chromosomes. Mutat Res.

[32] Shiraishi T, Druck T, Mimori K, Flomenberg J, Berk L, Alder H (2001). Sequence conservation at human and mouse orthologous common fragile regions, FRA3B/FHIT and Fra14A2/Fhit. Proc Natl Acad Sci USA.

[33] Glover TW, Wilson TE, Arlt MF (2017). Fragile sites in cancer: more than meets the eye. Nat Rev Cancer.

[34] Sozzi G, Veronese ML, Negrini M, Baffa R, Cotticelli MG, Inoue H (1996). The FHIT gene 3p14.2 is abnormal in lung cancer. Cell.

[35] Zack TI, Schumacher SE, Carter SL, Cherniack AD, Saksena G, Tabak B (2013). Pan-cancer patterns of somatic copy number alteration. Nat Genet.

[36] Somyajit K, Saxena S, Babu S, Mishra A, Nagaraju G (2015). Mammalian RAD51 paralogs protect nascent DNA at stalled forks and mediate replication restart. Nucleic Acids Res.

[37] Takata M, Sasaki MS, Tachiiri S, Fukushima T, Sonoda E, Schild D (2001). Chromosome instability and defective recombinational repair in knockout mutants of the five Rad51 paralogs. Mol Cell Biol.

[38] Serra H, Da Ines O, Degroote F, Gallego ME, White CI (2013). Roles of XRCC2, RAD51B and RAD51D in RAD51-independent SSA recombination. PLoS Genet.

[39] Termolino P, Cremona G, Consiglio MF, Conicella C (2016). Insights into epigenetic landscape of recombination-free regions. Chromosoma.

[40] Vincenten N, Kuhl LM, Lam I, Oke A, Kerr AR, Hochwagen A et al. The kinetochore prevents centromere-proximal crossover recombination during meiosis. *Elife.* 2015;4:e10850. 10.7554/eLife.10850 PMC4749563 26653857

[41] Jaco I, Canela A, Vera E, Blasco MA (2008). Centromere mitotic recombination in mammalian cells. J Cell Biol.

[42] Gisselsson D, Pettersson L, Hoglund M, Heidenblad M, Gorunova L, Wiegant J (2000). Chromosomal breakage-fusion-bridge events cause genetic intratumor heterogeneity. Proc Natl Acad Sci USA.

[43] Garsed DW, Marshall OJ, Corbin VD, Hsu A, Di Stefano L, Schroder J (2014). The architecture and evolution of cancer neochromosomes. Cancer Cell.

[44] Marotta M, Onodera T, Johnson J, Budd GT, Watanabe T, Cui X (2017). Palindromic amplification of the ERBB2 oncogene in primary HER2-positive breast tumors. Sci Rep.

[45] Dillon LW, Pierce LC, Ng MC, Wang YH (2013). Role of DNA secondary structures in fragile site breakage along human chromosome 10. Hum Mol Genet.

[46] Helmrich A, Ballarino M, Tora L (2011). Collisions between replication and transcription complexes cause common fragile site instability at the longest human genes. Mol Cell.

[47] Alzu A, Bermejo R, Begnis M, Lucca C, Piccini D, Carotenuto W (2012). Senataxin associates with replication forks to protect fork integrity across RNA-polymerase-II-transcribed genes. Cell.

[48] Paeschke K, Capra JA, Zakian VA (2011). DNA replication through G-quadruplex motifs is promoted by the Saccharomyces cerevisiae Pif1 DNA helicase. Cell.

[49] Leon-Ortiz AM, Svendsen J, Boulton SJ (2014). Metabolism of DNA secondary structures at the eukaryotic replication fork. DNA Repair.

[50] Orii KE, Lee Y, Kondo N, McKinnon PJ (2006). Selective utilization of nonhomologous end-joining and homologous recombination DNA repair pathways during nervous system development. Proc Natl Acad Sci USA.

[51] Rickert RC, Roes J, Rajewsky K (1997). B lymphocyte-specific, cre-mediated mutagenesis in mice. Nucleic Acids Res.

[52] Robbiani DF, Bothmer A, Callen E, Reina-San-Martin B, Dorsett Y, Difilippantonio S (2008). AID is required for the chromosomal breaks in c-myc that lead to c-myc/IgH translocations. Cell.

