# Supplementary material for: Visualizing locus-specific sister chromatid exchange reveals differential patterns of replication stress-induced fragile site breakage
Source: Oncogene. 2019 Oct 21;39(6):1260–72. doi: 10.1038/s41388-019-1054-5 (PMC7002298; doi:10.1038/s41388-019-1054-5)
Supplement: Supplementary file 1 — Supplementary Figure Legends [file 41388_2019_1054_MOESM1_ESM.docx]

**Supplementary figure legends**

**Supplementary Figure 1: SCE-FISH measures successful recombination-mediated repair at endogenous genomic loci. a)** Representative chromosome showing a “twist” in the chromosome arms highlighted by BrdU staining. BrdU is visualized in cyan, telomeres in red, and DAPI in greyscale. Representative chromosome is from cells treated with1 μM ATRi. **b)** Quantitation of spontaneous breaks at *GIMAP* in *Xrcc2^f/f^* cells, 8% of breaks occur at the *GIMAP* probe. **c)** Fraction of spontaneous DNA breaks at *GIMAP*.

**Supplementary Figure 2: Exposure to 1 μM** **ATR inhibitor induces DNA breaks and SCE events at ERFS and CFS. a)** Number of breaks per metaphase in WT and *Xrcc2^f/f^* cells exposed to1 μM ATRi. **b)** Percent of metaphases (cells) with a break at the specified locus in WT and *Xrcc2^f/f^* cells. **c)** Number of SCEs per metaphase at the sites of interest in WT and *Xrcc2^f/f^* cells. **d)** Percent of metaphases with an SCE at ERFSs, CFSs and cold sites. Error bars show the standard error of mean (SEM) from 3 independent experiments. Statistics: **p* < 0.05 comparing untreated and ATRi-treated cells for each genotype; #*p* < 0.05 comparing WT and *Xrcc2^f/f^* cells treated with ATR inhibitor.

**Supplementary** **Figure 3: Exposure to 0.4 μM APH induces DNA breaks and SCE events at ERFSs and CFSs. a)** Number of breaks per metaphase at the specified locus in WT and *Xrcc2^f/f^* cells. **b)** Percent of metaphases with a break at the specified locus in WT and *Xrcc2^f/f^* cells. **c)** Number of metaphases with an SCE at the specified locus in WT and *Xrcc2^f/f^* cells. **d)** Percent of metaphases with an SCE at the specified locus in WT and *Xrcc2^f/f^* cells. Error bars show the SEM from 3 independent experiments. Statistics: **p* < 0.05 comparing untreated and 0.4 uM APH-treated cells of each type; #*p* < 0.05 comparing 0.4 uM APH-treated cells of different genotypes. **e)** Number of breaks per metaphase in *Xrcc2^f/f^* cells infected with either empty vector (MIGR1-EV) or XRCC2 (MIGR1-X2) exposed to 0.4 μM APH. **f)** Frequency of breaks at *GIMAP* and *IMMP2L* in *Xrcc2^f/f^* cells infected with MIGR1-EV or MIGR1-X2 exposed to 0.4 μM APH. Error bars show the SEM from 2 independent infection experiments. APH was added to cells ~19 hours before sorting for panels **e** and **f**. Statistics: **p* < 0.05 comparing untreated and 0.4 uM APH-treated cells of each type; ^*p* < 0.0562 comparing 0.4 uM APH-treated cells infected with MIGR1-EV or MIGR1-X2.

**Supplementary Figure 4: Crossovers are suppressed at centromere-proximal fragile sites. a)** Frequency of SCE formation at *IKZF1* compared to the ERFS *GIMAP* and *BCL2*, the CFSs *IMMP2L* and *FHIT*, and two cold sites in WT cells. **b)** Frequency of DNA damage at *IKZF1* compared to ERFSs, CFSs and cold sites in WT cells. Error bars show the SEM from 3 independent experiments. **c)** Example of SCE at *FHIT*. **d)** Example of SCE at *IKZF1* showing SCE events are easily visualized even in highly compacted chromosomes. Representative chromosomes are from cells treated with1 μM ATRi. For c and d, probe for *FHIT* and *IKZF1* shown in green, telomeres in red, BrdU in cyan, and DAPI in greyscale.

**Supplementary Figure 5. SCE-FISH reveals CFS breakage and repair in primary human lymphocytes. a)** Number of breaks per metaphase at ERFSs, CFSs, and cold sites in human PBMCs exposed to 0.4 μM APH. **b)** Percent of metaphases with a break at ERFSs, CFSs and cold sites. **c)** Number of SCEs per metaphase at ERFSs, CFSs and cold sites. **d)** Percent of metaphases with an SCE at ERFSs, CFSs, and cold sites in human PBMCs exposed to 0.4 μM APH. Statistics: **p* < 0.05 comparing untreated and 0.4 μM APH-treated cells.
