# Supplementary figures and images for: Visualizing locus-specific sister chromatid exchange reveals differential patterns of replication stress-induced fragile site breakage
Source: Oncogene. 2019 Oct 21;39(6):1260–72. doi: 10.1038/s41388-019-1054-5 (PMC7002298; doi:10.1038/s41388-019-1054-5)

Supplementary Figure 1

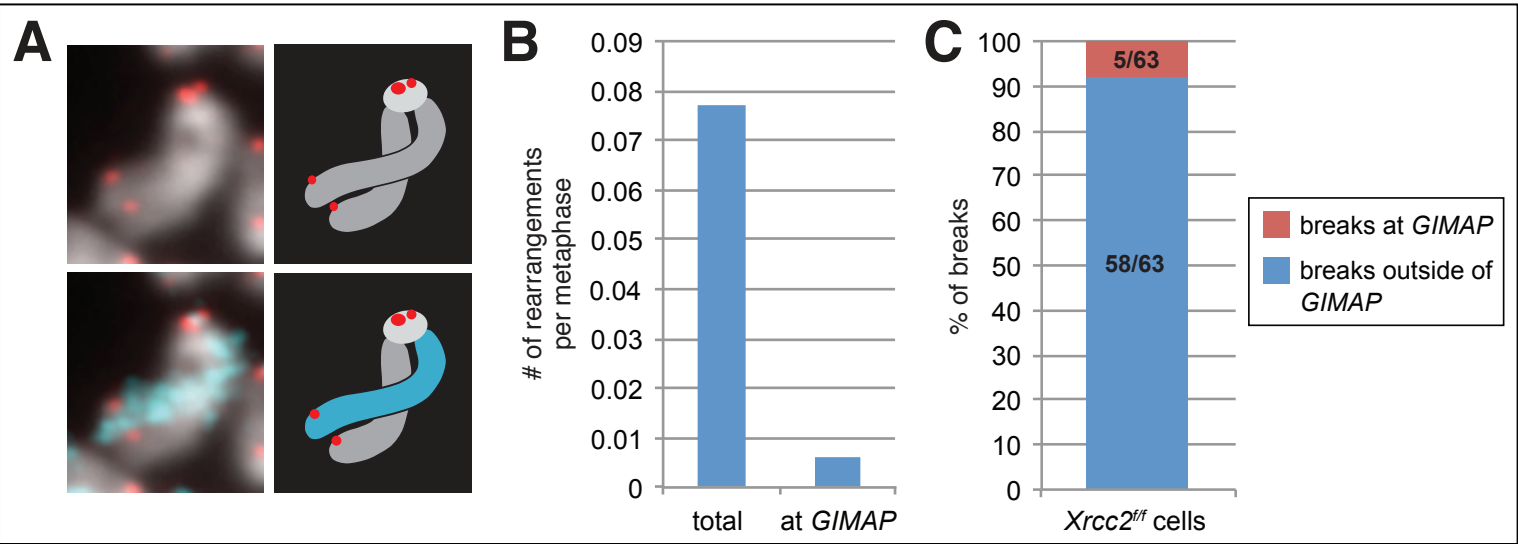

Supplement: Supplementary file 2 — Supplementary Figure 1 [file 41388_2019_1054_MOESM2_ESM.pdf]

Supplementary Figure 2

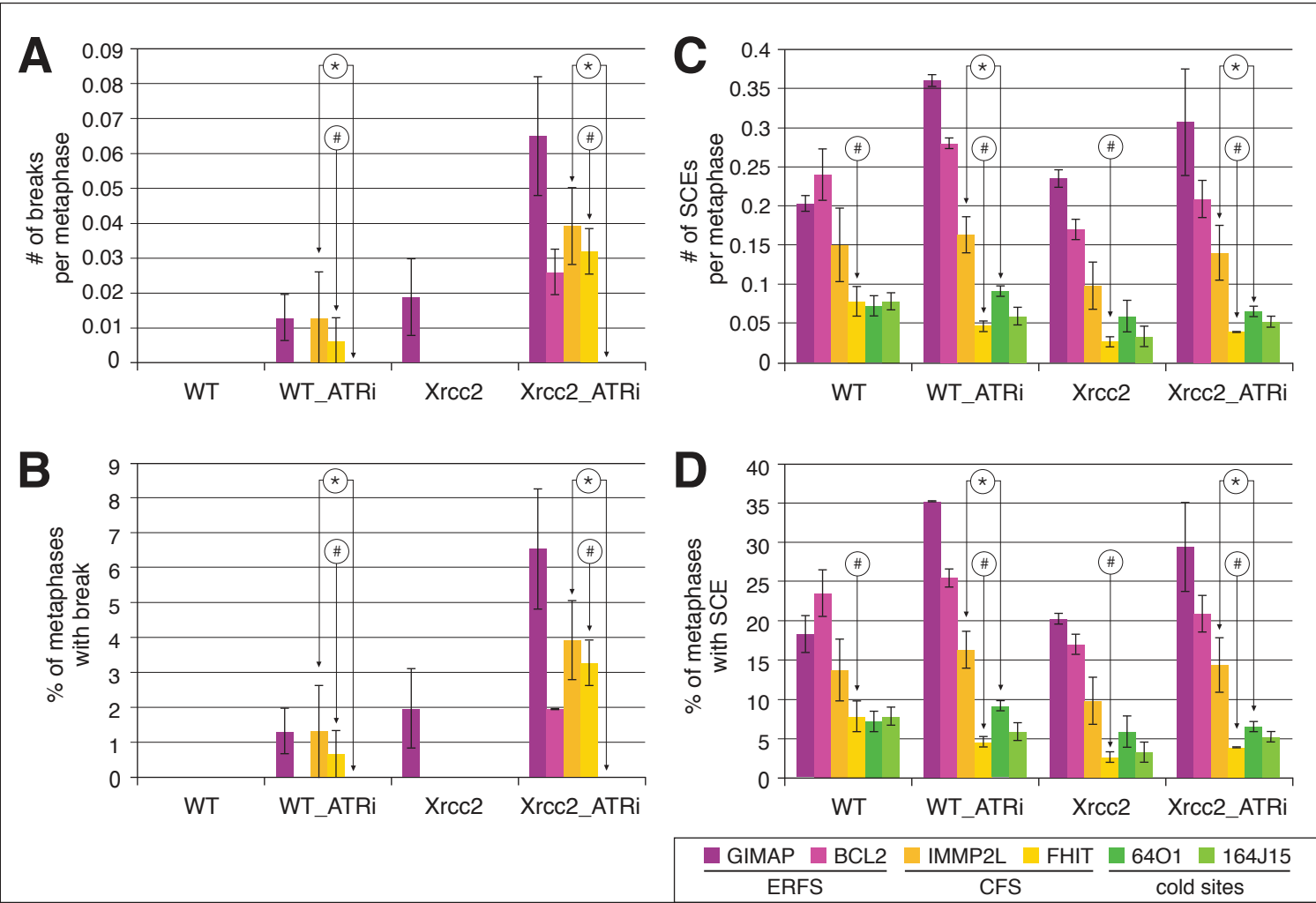

Supplement: Supplementary file 3 — Supplementary Figure 2 [file 41388_2019_1054_MOESM3_ESM.pdf]

Supplementary Figure 3

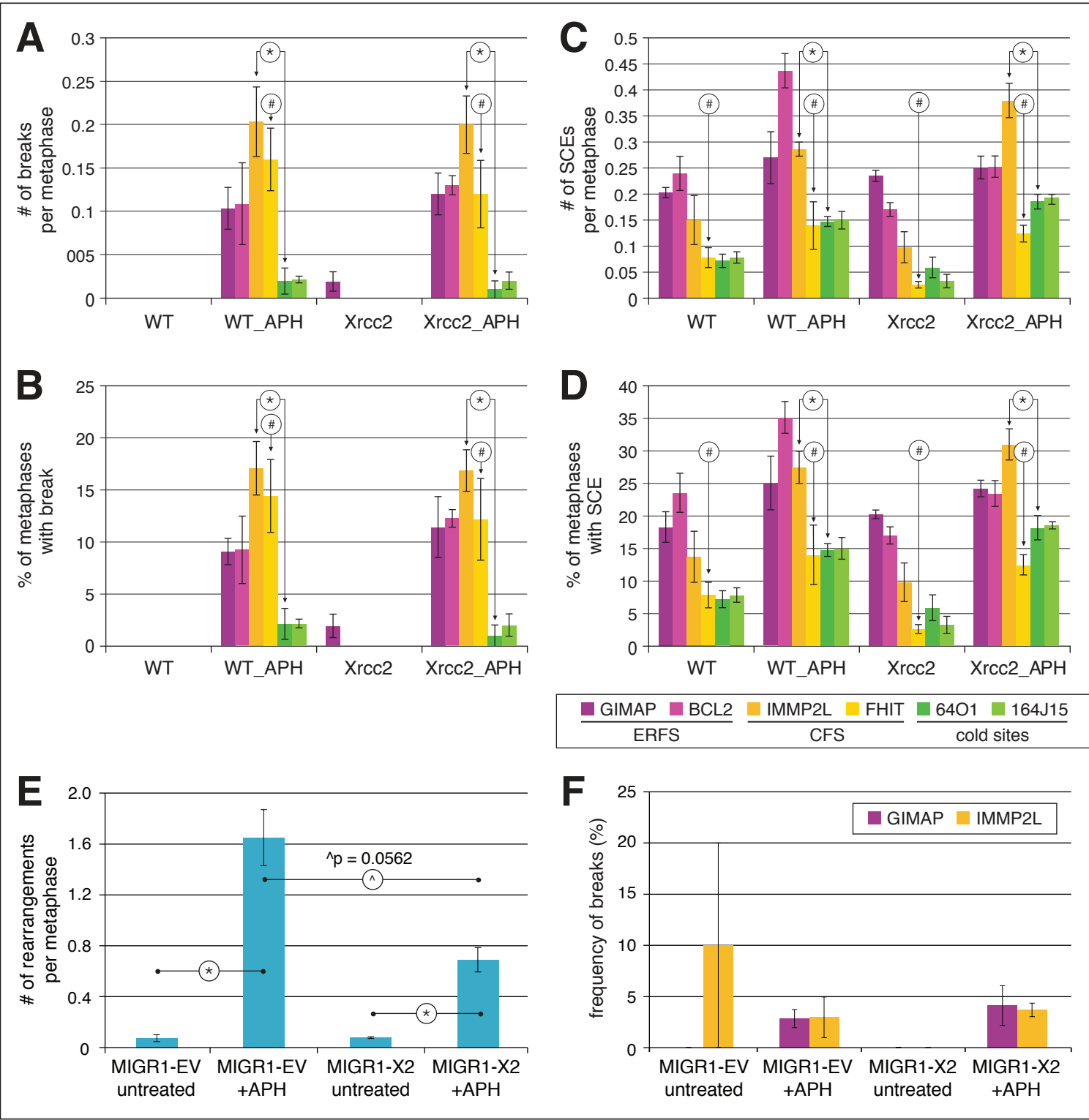

Supplement: Supplementary file 4 — Supplementary Figure 3 [file 41388_2019_1054_MOESM4_ESM.pdf]

Supplementary Figure 4

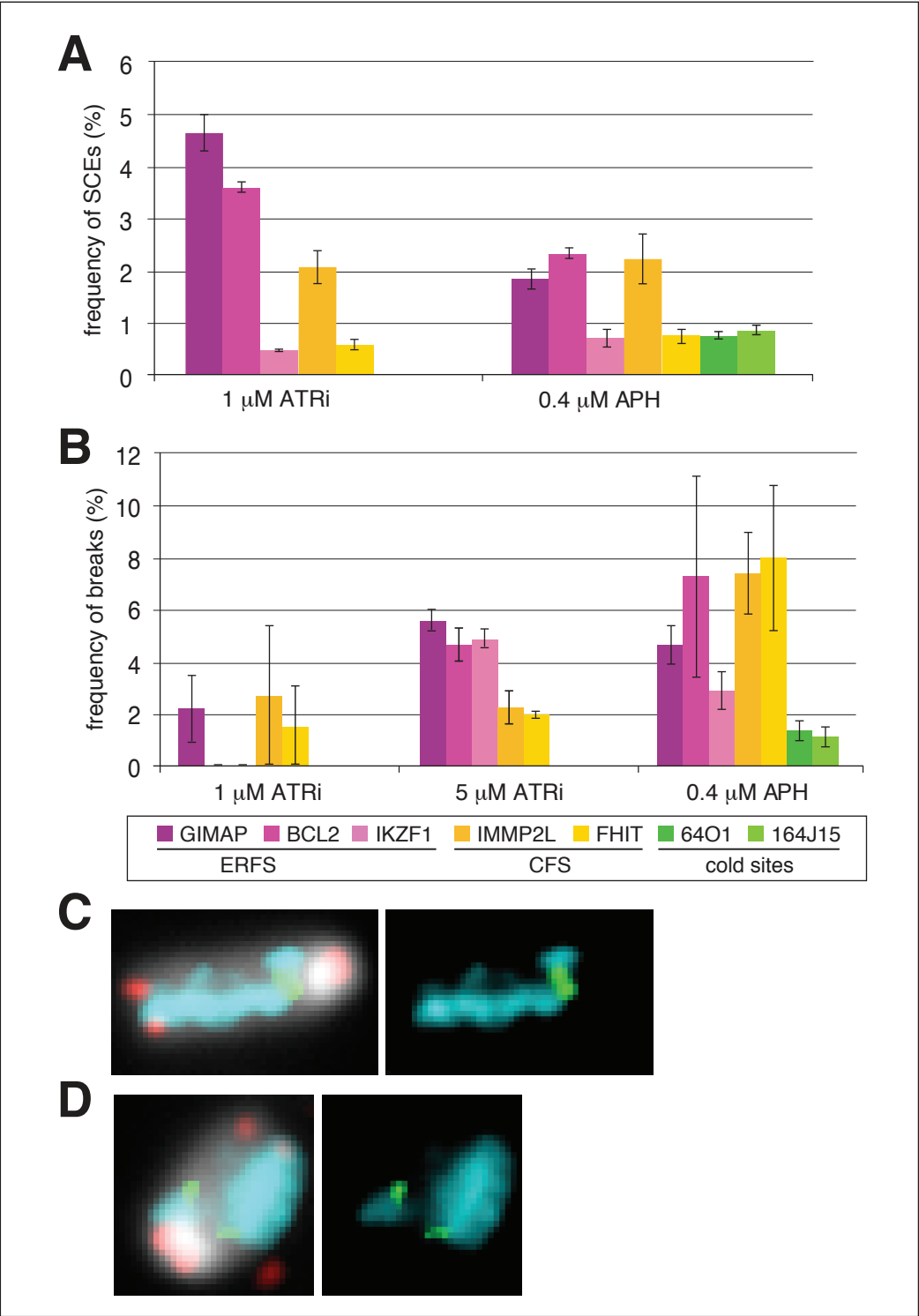

Supplement: Supplementary file 5 — Supplementary Figure 4 [file 41388_2019_1054_MOESM5_ESM.pdf]

Supplementary Figure 5

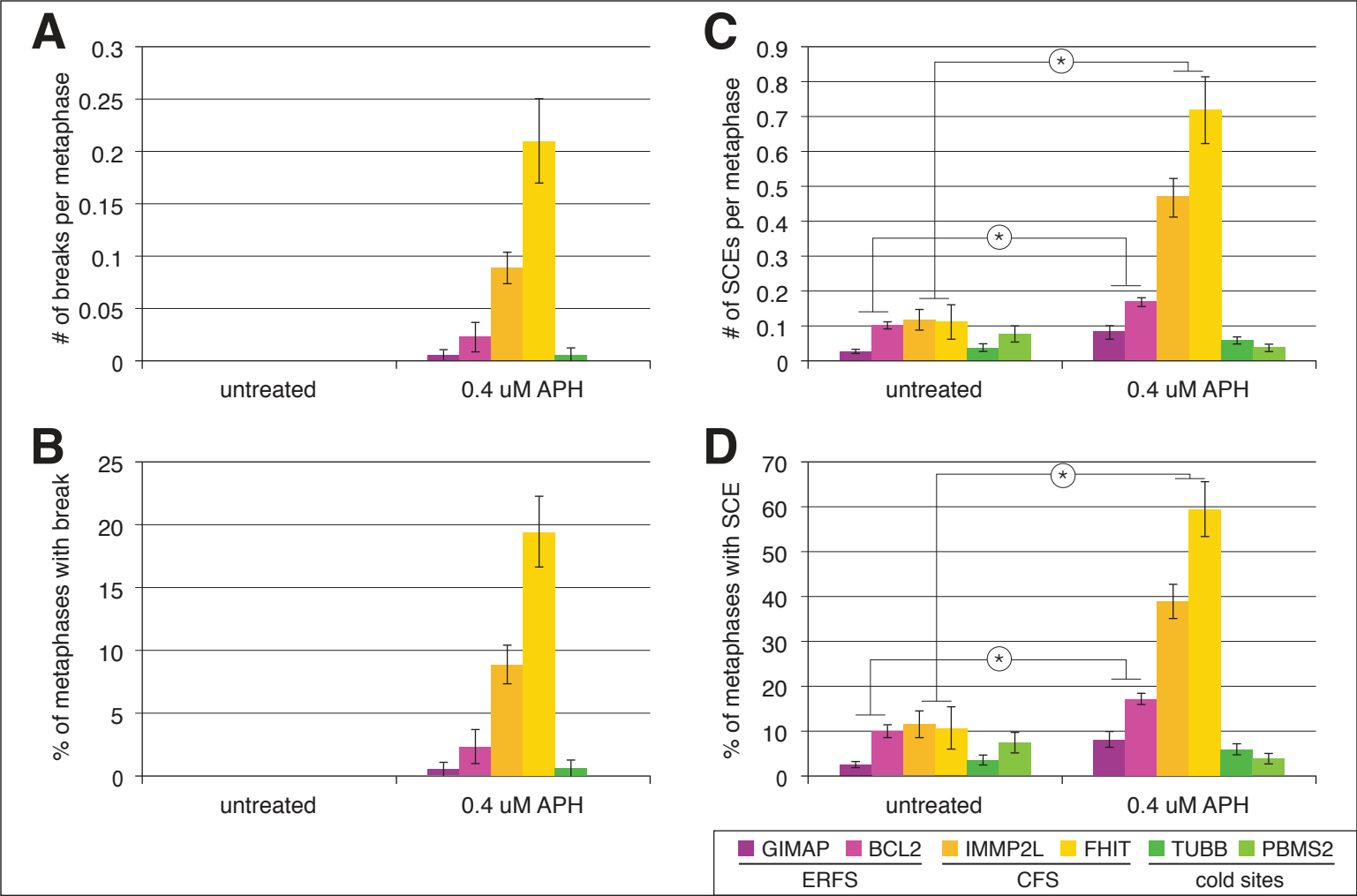

Supplement: Supplementary file 6 — Supplementary Figure 5 [file 41388_2019_1054_MOESM6_ESM.pdf]
